# Crizotinib inhibits NF2-associated schwannoma through inhibition of focal adhesion kinase 1

**DOI:** 10.18632/oncotarget.10248

**Published:** 2016-06-23

**Authors:** Scott Troutman, Susana Moleirinho, Smitha Kota, Kendall Nettles, Mohammad Fallahi, Gary L. Johnson, Joseph L. Kissil

**Affiliations:** ^1^ Department of Cancer Biology, The Scripps Institute, Jupiter, FL, 33458, USA; ^2^ Department of Informatics Core, The Scripps Institute, Jupiter, FL, 33458, USA; ^3^ Department of Pharmacology, University of North Carolina, Chapel Hill, NC, 27599, USA

**Keywords:** neurofibromatosis, NF2, signal transduction, crizotinib, FAK

## Abstract

Neurofibromatosis type 2 (NF2) is a dominantly inherited autosomal disease characterized by schwannomas of the 8th cranial nerve. The *NF2* tumor suppressor gene encodes for Merlin, a protein implicated as a suppressor of multiple cellular signaling pathways. To identify potential drug targets in NF2-associated malignancies we assessed the consequences of inhibiting the tyrosine kinase receptor MET. We identified crizotinib, a MET and ALK inhibitor, as a potent inhibitor of *NF2*-null Schwann cell proliferation *in vitro* and tumor growth *in vivo*. To identify the target/s of crizotnib we employed activity-based protein profiling (ABPP), leading to identification of FAK1 (PTK2) as the relevant target of crizotinib inhibition in *NF2*-null schwannoma cells. Subsequent studies confirm that inhibition of FAK1 is sufficient to suppress tumorigenesis in animal models of NF2 and that crizotinib-resistant forms of FAK1 can rescue the effects of treatment. These studies identify a FDA approved drug as a potential treatment for NF2 and delineate the mechanism of action in *NF2*-null Schwann cells.

## INTRODUCTION

Neurofibromatosis type 2 (NF2) is a dominantly inherited autosomal disease (affecting 1 in 30,000), attributed to the loss-of-heterozygosity (LOH) of the *NF2* gene. The disease is characterized mainly by development of Schwann cell tumors of the eighth cranial nerve. Mutations and loss of heterozygosity (LOH) of the *NF2* locus have also been detected at high frequency in various tumors of the nervous system, including sporadic schwannomas, meningiomas and ependymomas [1]. The *NF2* tumor suppressor gene encodes a 69-kDa protein called Merlin (Moesin, ezrin, and radixin like protein), implicated in the regulation of a number of the Rac1, Ras/MAPK, mTOR and Hippo signaling pathways [2]. Previous studies have demonstrated that Merlin interacts with CD44 and inhibits signaling through the MET proto-oncogene, a receptor tyrosine kinase [3]. In addition, analysis of *NF2*-null vestibular schwannomas demonstrated dysregulation of MET expression and other associated genes [4]. Moreover, several studies have demonstrated that Merlin functions as an inhibitor of the group I p21-activated kinases (PAKs), by direct inhibition and by inhibition of the small G-protein Rac1, a direct upstream activator of the PAKs [5–8]. Previous studies have shown that the p21-activated kinase 1 (PAK1) activates MET signaling and that disruption of this activity inhibits PAK1-driven anchorage-independent cell growth [9]. This is especially significant in the case of NF2, as previous work has established the PAKs as necessary effectors and targets for inhibition in NF2 [6, 10]. These findings implicate MET as a potential target for therapeutic inhibition in the context of *NF2* loss. We therefore set out to determine whether MET inhibition would be beneficial in a model of NF2-associated schwannoma.

Towards this goal we employed pharmacologic and genetic approaches to inhibit MET and identified the FDA approved drug crizotinib (PF-2341066), as a potent inhibitor of tumor progression and cellular proliferation of *NF2*-null Schwann cells. Moreover, our studies establish the inhibition of FAK1 (PTK2) as the primary mechanism of action for crizotinib in these cells. As crizotinib is well tolerated and is currently being evaluated in clinical trials in pediatric settings, our findings provide a strong rationale for assessing this drug in NF2 patients.

## RESULTS

### Crizotinib inhibits schwannoma cell proliferation and tumor formation *in vivo*

**Figure 1:**
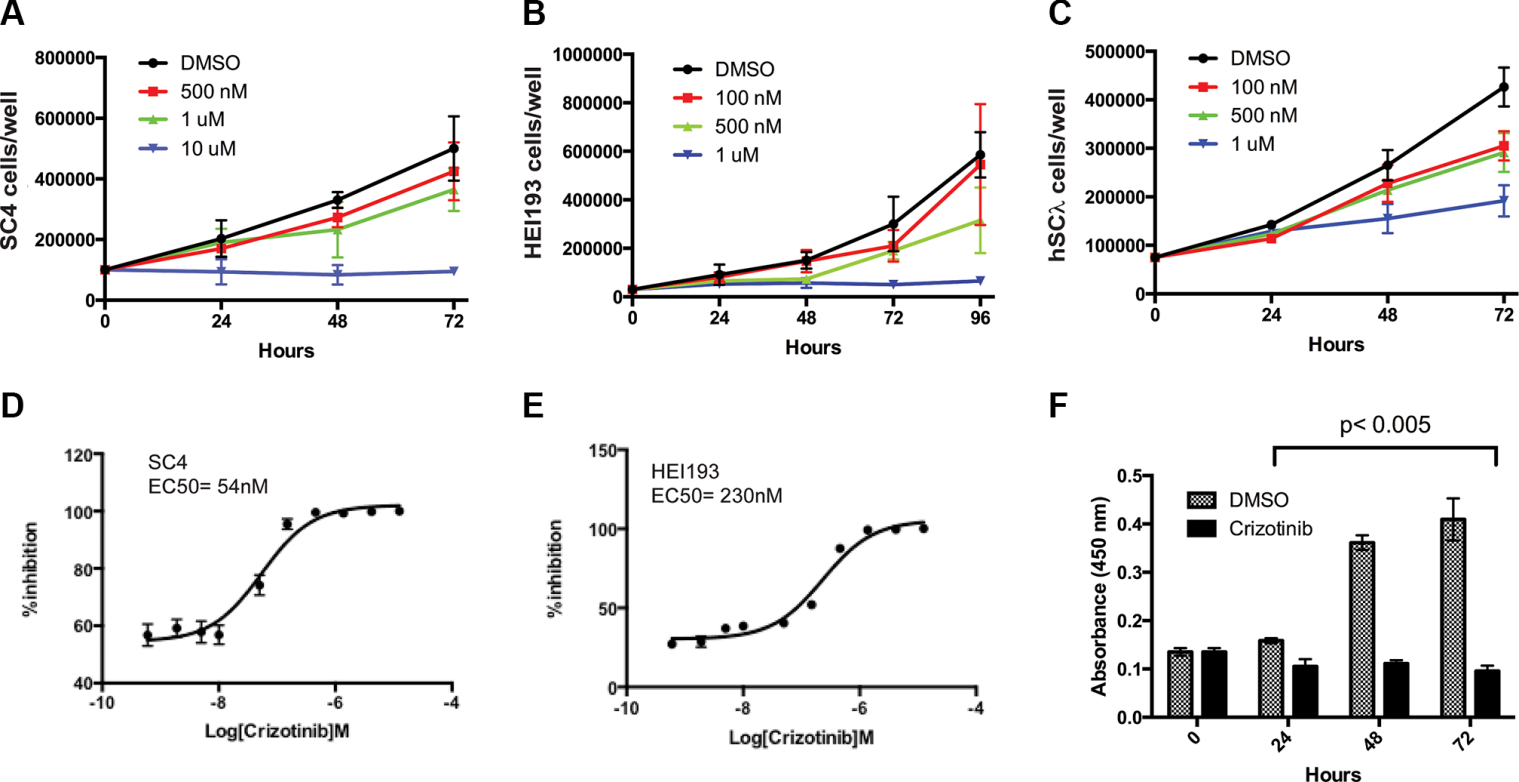
Impact of crizotinib treatment on *NF2*-null schwann cell proliferation (**A**) SC4, (**B**) HEI193 or (**C**) hSC2λ-shNF2 cells treated with crizotinib at the indicated concentrations or with 25% DMSO control, daily for 3 days. (**D–E**) 10-point dose response curves assessing viability of SC4 or HEI193 cells treated with Crizotinib at the indicated concentrations. (**F**) The proliferation of SC4 cells was assessed by BrdU incorporation at 24, 48 and 72 hours post crizotinib treatment (1 μM) compared to control (DMSO). In all counting experiments cell numbers were scored daily and each time point was done in triplicate. The data shown is the mean of 3 independent experiments. Error bars = SD.

As previous studies implicated signaling downstream of MET as a potential therapeutic target in vestibular schwannoma [3, 4], we assessed the consequences of inhibiting MET in *NF2*-null Schwann cells. HEI193 cells (*NF2*-null, derived from a schwannoma of an NF2 patient), SC4 cells (*Nf2*-null mouse cells) and hSC2λ-shNF2 cells (hSC2λ cells – immortalized normal human Schwann cells with stable knockdown of NF2) were treated with crizotinib (PF-2341066), a FDA approved drug that is a potent inhibitor of MET, ALK and Ros-1 [11]. Treatment of these cells resulted in a dose-dependent reduction in cell numbers over a period of 72 hours (Figure 1A–1C). To more accurately assess the inhibitory activity of crizotinib, we performed dose-response studies and established the EC_50_s as 54 nM and 230 nM in SC4 or HEI193 cells, respectively (Figure 1D–1E). To determine if the effects of crizotinib are due to reduced cell proliferation or increased cell death rates, we assessed BrdU incorporation in treated SC4 cells. BrdU incorporation was significantly reduced over 72-hours, indicating that crizotinib has a strong effect on cellular proliferation (Figure 1F). To assess the potential effects of crizotinib on cell death, we examined the status of a number of apoptotic markers in the treated cells. We did not observe any differences in levels of cleaved Caspase-3, Caspase-7 and PARP between SC4 cells treated with crizotinib or vehicle only (not shown). These data suggest the effects of crizotinib are mediated primarily through inhibition of cell proliferation.

**Figure 2:**
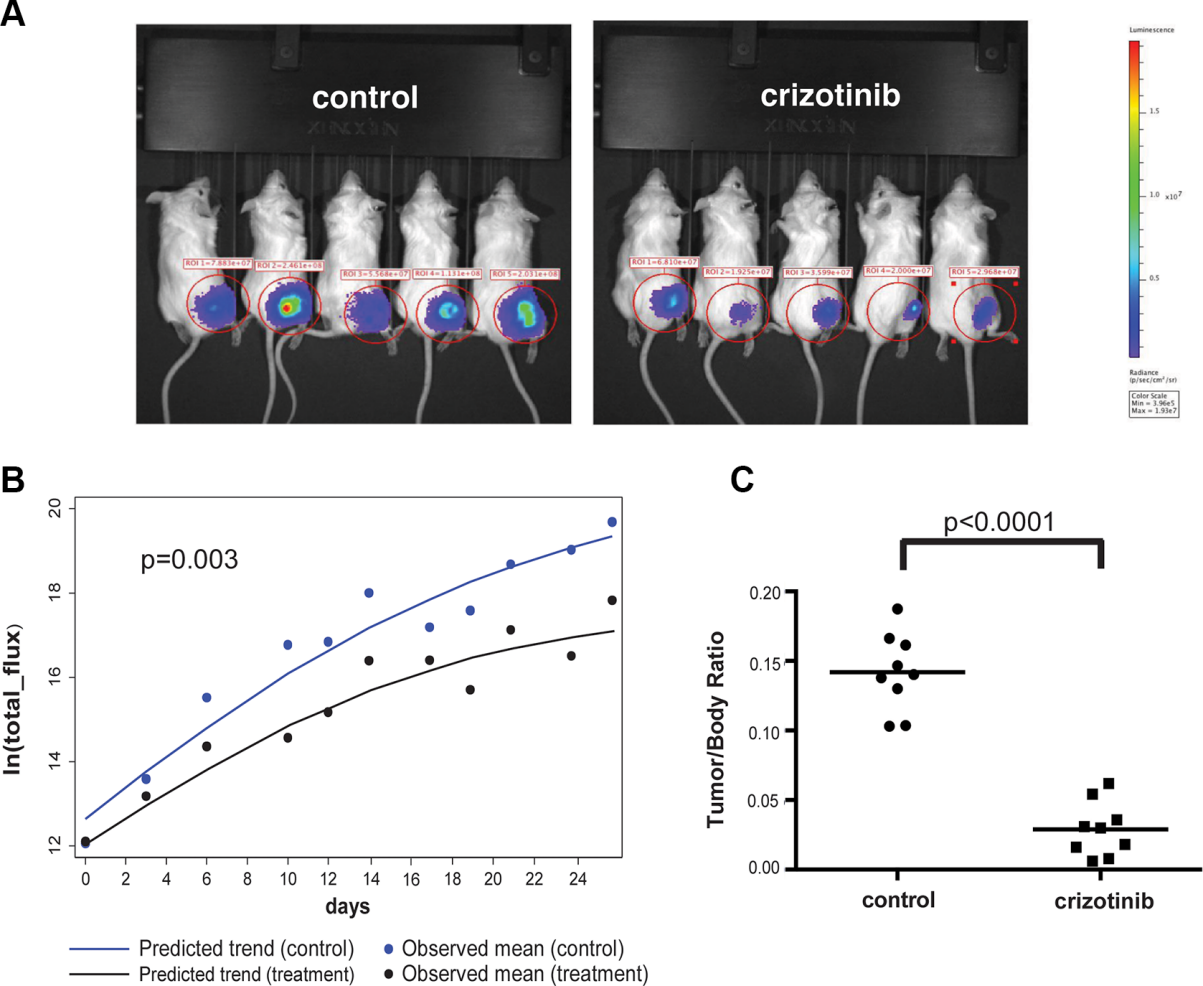
Crizotinib inhibits tumor growth *in vivo* (**A**) Representative images from bioluminescence imaging (BLI) of mice carrying orthotopic tumors treated with crizotinib (50 mg/kg) or vehicle control (25% DMSO) at day 14 of treatment. NOD/SCID mice were injected intraneurally with 5 × 10^4^ SC4/pLuc-mCherry cells and were enrolled into treatment after 10 days. Mice were treated daily for 25 days and imaged every 3 days to follow tumor development. (**B**) Quantitative analysis of the flux reading from treated cohorts. A mixed-effect model analysis indicated that the speed of tumor growth in treatment group is significantly slower than that in control group (*p* = 0.003). (**C**) Distribution of tumor/body weight ratio in the cohorts treated with crizotinib or vehicle control. The results of *t*-test with equal variances show that the crizotinib-treated group has significant lower average tumor weight than that observed in control group (*p* < 0.0001). For the *in vivo* experiments the *N* = 9 in each cohort.

To assess if crizotinib can inhibit tumor growth *in vivo*, we employed an orthotopic model of NF2 that recapitulates the tumor microenvironment of schwannomas, by injection of luciferase-tagged *NF2*-null SC4 cells into a myelinated nerve [12, 13]. In this model, tumors are first allowed to form and only then treatment is commenced. Beginning at 3 days post surgery, tumor progression was monitored every three days by bioluminescence imaging (BLI) and total flux counts were recorded for each animal. Ten days post injection, similar flux readings for all animals were validated and animals were then enrolled randomly into control (vehicle only) or drug-treated cohorts and were treated (100 mg/kg, IP, once daily) for a period of 24 days. Analysis of the flux reading for the animals in the cohorts indicated the trends of tumor growth are significantly different between the cohorts and that crizotinib treated mice displayed a significantly slower tumor growth rate (Figure 2A–2B). After 24 days of treatment the animals were sacrificed and the tumors removed and weighed. Comparison of the cohorts demonstrates a significantly lower average tumor weight in the crizotinib-treated group compared to control group (Figure 2C). Taken together, these data demonstrate that crizotinib has significant anti-proliferative activity against *NF2*-null Schwann cells *in vitro* and anti-tumor activity *in vivo.*

### MET is dispensable for *NF2*-null schwann cell proliferation

To determine whether the effects of crizotinib are mediated through inhibition of MET, we employed pharmacological and genetic approaches to target MET more precisely. HEI193 or SC4 cells were treated with capmatinib (INC280), a potent MET inhibitor with reported average IC_50_ of 0.13 nmol/L. INC280 is highly selective towards MET and at 2 μmol/L exhibited 30% or less inhibition against a panel of 57 structurally diverse kinases [14]. Treatment of SC4 or HEI193 cells with doses of up to 10 μM and up to 72 hours had negligible effects on the proliferation of treated cells compared to controls (Supplementary Figure S1A–1B). To assess the requirement for MET directly, genome editing using the CRISPR/Cas9 system was used to inactivate both *MET* alleles in SC4 cells, generating SC4^MET(−)^ cells (Supplementary Figure S1C). Inactivation of *MET* had a minimal effect on the proliferation of SC4^MET(−)^ cells compared to controls (Supplementary Figure S1D). Similar results were obtained using 2 independent siRNA oligos to knockdown the expression of MET (Not shown). Finally, treatment of SC4^MET(−)^ cells with crizotinib resulted in nearly identical activity compared to SC4 cells, suggesting the activity of crizotinib was not mediated through MET inhibition (Supplementary Figure S1D).

### Crizotinib inhibits a broad spectrum of kinases in *NF2*-null schwannoma cells

**Figure 3:**
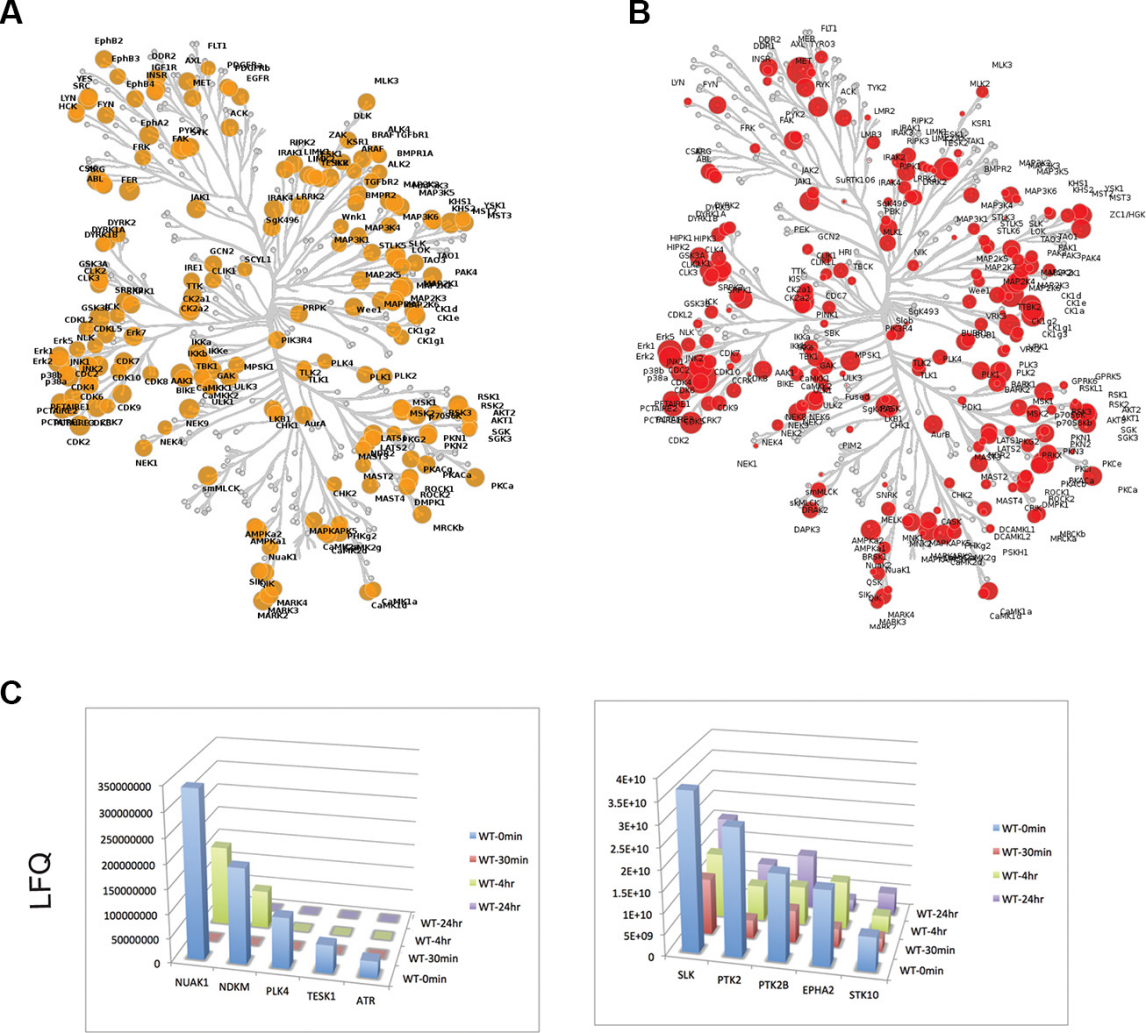
Expression and activity based profiling of the kinome in *NF2*-null schwann cells (**A**) Untreated SC4 cells were extracted for RNA and analyzed by sequencing. The expressed kinases (> 1 RPKM) are indicated in yellow on the dendogram, with the size of each circle representing the log_2_ transformed RPKM value. (**B**) Untreated SC4 cells were extracted for protein and the activity-based profile of the kinome was assessed by binding to a multiplexed inhibitor bead matrix, followed by elution of bound proteins and identification/quantification by label-free mass spectrometry. The levels of each kinase identified are indicated in red on the dendogram, with the size of each circle representing the log_2_ transformed quantification value. (**C**) Time-dependent activity-based profiles for kinases that met the selection criteria. SC4 cells were treated with 10 μM crizotinib and harvested at 0, 30′, 4 h and 24 h post treatment. LFQ = Label-Free quantification values.

As the anti-proliferative effects of crizotinib on SC4 and HEI193 do not appear to be mediated through inhibition of MET, we applied a combination of unbiased approaches to identify the relevant targets. First, we examined the expression of kinases in SC4 cells through unbiased RNA sequencing (RNA-Seq) reads. The normalized read-counts of all annotated kinases revealed a broad pattern of expression of kinases from all families. Interestingly, the typical targets of crizotinib ALK and Ros-1 do not appear to be expressed in SC4 cells (Figure 3A and Supplementary Table S1). Western blot analysis of protein extracts from SC4 and HEI193 to assess the expression of ALK and Ros-1 confirmed the lack of expression (not shown). To identify the kinases inhibited by crizotinib we employed activity-based protein profiling (ABPP). Briefly, we used an affinity column composed of multiple kinase inhibitors conjugated to Sepharose beads. This Multiplexed Inhibitor Bead (MIB) matrix provides the capability to capture > 85% of known protein kinases. The kinases were then eluted from the MIB matrix and identified and quantitated by mass spectrometry. This approach allowed us to compare multiple samples in a single run [15, 16]. Comparison of the kinases captured by the MIB matrix to the RNA-seq expression data indicates the matrix captured the vast majority of kinases expressed (at least at the transcriptional level) in the SC4 cells (Figure 3B).

We next treated SC4 cells with crizotinib and collected cells at 0′, 30′, 240′ and 24 hours post treatment. Protein extracts from the different time points were analyzed by ABPP, as described above, and levels of captured kinases were assessed over time. The binding to crizotinib should prevent binding of an inhibited kinase to the MIB matrix and therefore levels of the kinase in treated fractions will be decreased (Supplementary Table S2). We applied the following criteria to identify targets of crizotinib that might mediate the effects observed in SC4 cells: First, target displays > 2 fold reduction relative to untreated cells and, second, the reduction is sustained up to 24 hours. Applying these selection criteria resulted in the identification of 10 kinases: NUAK1, NDKM, PLK4, TESK1, ATR, SLK, PTK2, PTK2B, EPHA2 and STK10 (Figure 3C).

### Crizotinib's anti- proliferative activity is mediated by inhibition of FAK1/PTK2

**Figure 4:**
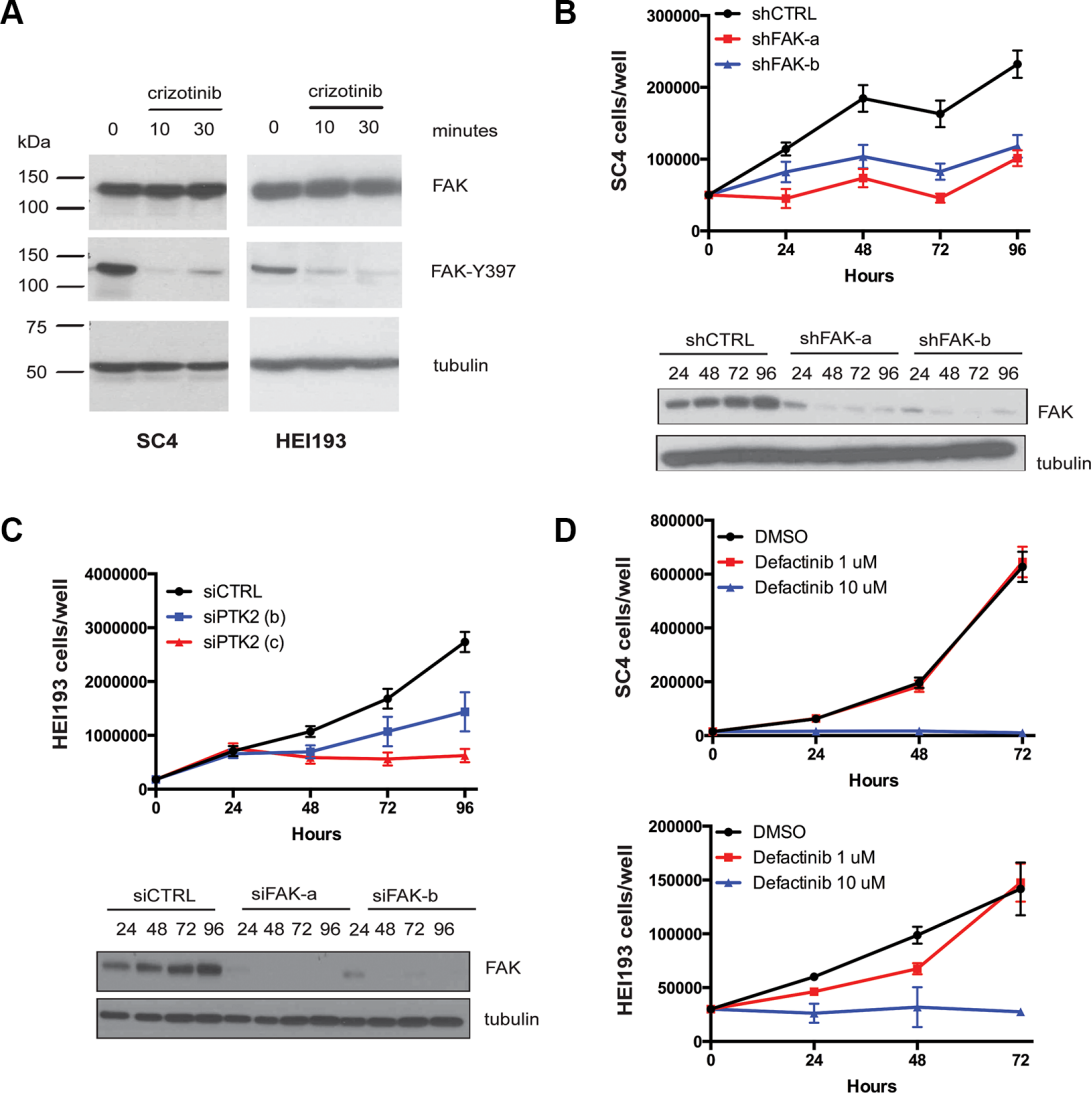
FAK1 is inhibited by crizotinib and required for proliferation of *NF2*-null schwann cells (**A**) Western blot analysis of total FAK1 and p-FAKY^397^ in protein extracts prepared from SC4 and HEI193 cells treated with crizotinib (10 μM for 10′ and 30′). Vinculin was used as a loading control. (**B**) Cell number counts of SC4 cells and western blot analysis of total FAK1 expression in cells treated with 2 independent shRNAs against FAK1 (shFAK-a, shFAK-b) or scrambled control (shCTRL). (**C**) Cell number counts of HEI193 cells and western blot analysis of total FAK1 expression in cells treated with 2 independent shRNAs against FAK1 (siFAK-a, siFAK-b) or scrambled control (siCTRL). (**D**) SC4 or HEI193 cells treated with defactinib at the indicated doses or with DMSO control, daily for 3 days. In all counting experiments cell numbers were scored daily and each time point was done in triplicate. The data shown represent the mean of 3 independent experiments. Error bars = SD.

Although the ABPP identified at least ten potential candidates for crizotinib's activity, given the previous reports correlating NF2 deficiency to sensitivity of FAK1 inhibition [17, 18] we focused initially on FAK1 as the potential relevant crizotinib target in *NF2*-null schwannoma cells. To confirm that indeed crizotinib inhibits FAK1 in SC4 and HEI193 cells we treated the cells and assessed FAK1 activation by western blot analysis using a phospho-specific antibody against FAK-Y397, which is phosphorylated in activated FAK1. As expected, treatment with crizotinib resulted in decreased FAK-Y397 phosphorylation indicating it inhibits the activation of FAK1 in these cells (Figure 4A). To determine if FAK1 is required for proliferation of SC4 or HEI193 cells we employed shRNA or siRNA, respectively, to knockdown expression of FAK1 in these cells. Indeed, the knockdown of FAK1 significantly impaired the proliferation of these cells (Figure 4B–4C). In addition, we examined the effects of treating SC4 or HEI193 cells with small-molecule FAK1 inhibitors. Treatment of SC4 or HEI193 cells with defactinib (VS6303) or PND-1186 (VS-4718) significantly impaired cell proliferation in a dose-dependent manner (Figure 4D and not shown).

**Figure 5:**
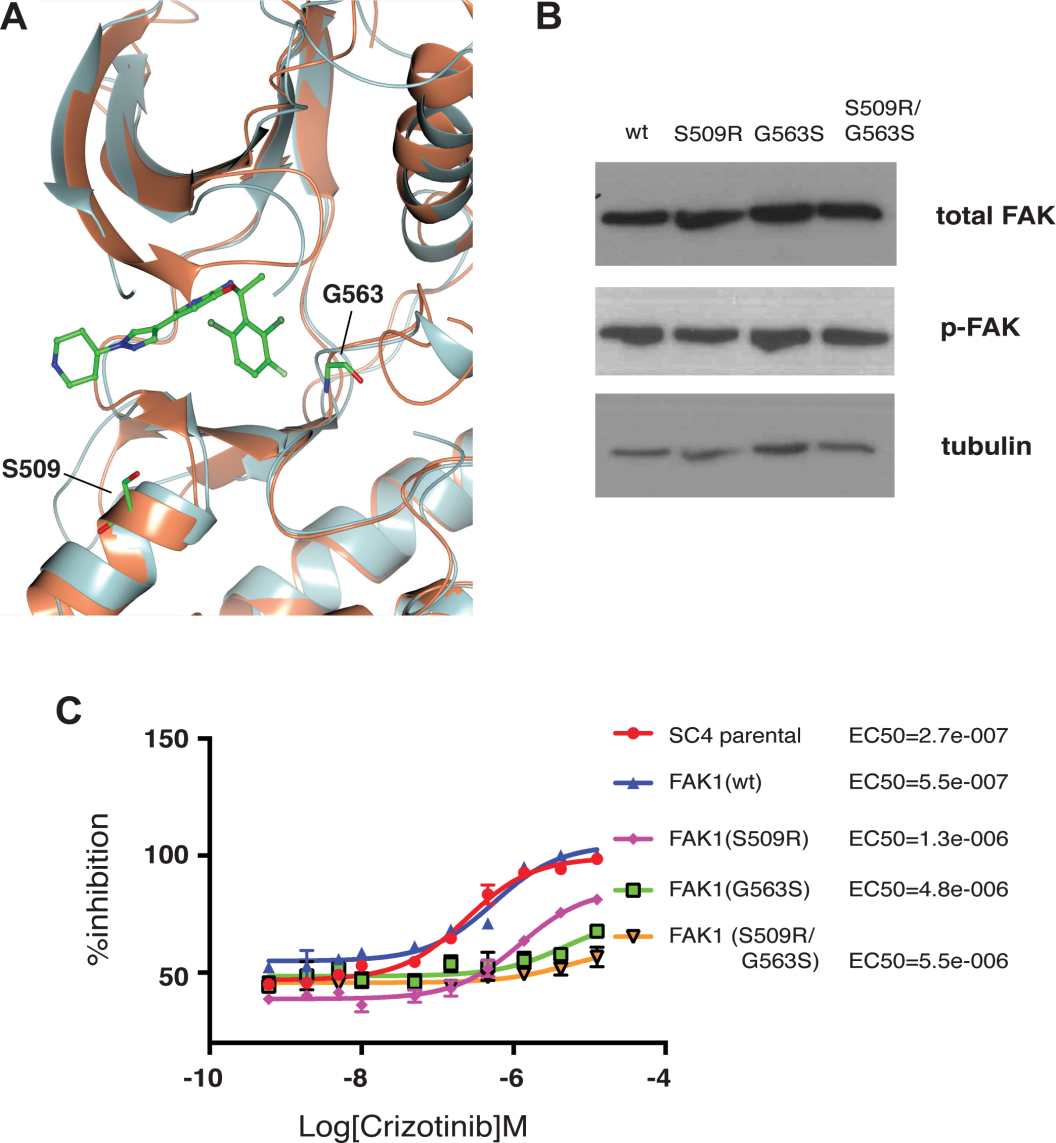
Crizotinib-resistant FAK1 mutants rescue proliferation of treated *NF2*-null schwann cells (**A**) Superimposition of the FAK1 (orange) and ALK (gray) kinase domains with crizotinib (ball and stick). Residues Glycine 509 (G509) and Serine 563 (S563) are highlighted (ball and stick). (**B**) Western blot analysis of the different FAK1 mutant expression in stably transfected SC4 cells. Vinculin was used as a loading control (**C**) 10-point dose response curves assessing EC50 of crizotinib in SC4 cells stably expressing crizotinib resistant FAK1 mutants. Calculated EC50 for each clone is indicated to the right. The data shown represents the mean of 3 independent experiments, each done in quadruplicate. Error bars = SD.

As the loss of function studies demonstrated crizotinib inhibits FAK1 in *NF2*-null Schwann cells and is required for the proliferation of these cells, we sought to confirm these observations through gain of function approaches. Towards this aim, we first identified ALK mutations previously shown to confer a decreased sensitivity to crizotinib and assessed their conservation in FAK1. Mutations at residue S1206, located at the solvent front of the kinase domain, and at G1269, located in proximity to the DFG motif, have been suggested to diminish the affinity of ALK to crizotinib and facilitate cell survival in response to treatment [19, 20]. Both S1206 and G1269 are located in regions of ALK that are highly conserved in FAK1 with the equivalent residues at Serine 509 and Glycine 563, respectively (Figure 5A). We thus generated stable SC4 cells expressing FAK1^wt^, FAK1^S509R^, FAK1^G563^ and FAK1^S509R/G563S^ and assessed the expression levels and activity of the different FAK1 alleles. The expression levels of FAK1 are similar for the wild type and mutated alleles as are the levels of activation, as determined by phosphorylation of the Y397 autophosphorylation site (Figure 5B). To determine whether the mutated alleles confer resistance to crizotinib we assessed the EC_50_s for SC4 cells stably expressing the different alleles. In a similar manner to what has been previously reported for ALK, the FAK1 S509R, G563S and S509R/G563S alleles conferred resistance to crizotinib treatment. While expression of wild type FAK1 increased the EC_50_ by 2-fold, the expression of the mutated FAK1 alleles shifted the EC_50_ by an order of magnitude (Figure 5C). Thus, the results from both loss- and gain-of-function experiments strongly implicate FAK1 inhibition as the main mechanism through which crizotinib mediates its effects in *NF2*-null schwannoma cells.

## DISCUSSION

In spite of more than 2 decades of research into the molecular basis of NF2, treatment options are still lacking. Current treatment options are anti-symptomatic, are limited to radiation or surgery and associated with severe morbidity. In an effort to identify suitable therapeutic targets, we initially focused on the MET tyrosine kinase inhibitor, based on previous reports implicating it as a potential target [3, 4, 9]. While our findings indicate MET is mostly dispensable for the proliferation of *NF2*-null Schwann cells, our efforts identified crizotinib (Xalkori^®^- Pfizer) as a having significant anti-proliferative activity both *in vitro* and *in vivo*. Crizotinib was developed as an ATP-competitive inhibitor of the anaplastic lymphoma kinase (ALK) and the MET/hepatocyte growth factor receptor (HGFR) [11, 21]. However, it has been shown to bind and inhibit multiple additional targets including the ROS1 and RON receptor tyrosine kinases [22–24].

As the primary targets of crizotinib inhibition do not appear to be expressed in *NF2*-null Schwann cells, we employed ABPP to identify 10 kinases that appear to be significantly inhibited by crizotinib. Although it is possible that any of these kinases, either alone or in combination, represents the target through which crizotinib mediates its anti-tumor effects, the recently reported association between NF2-status and sensitivity to FAK1 inhibition (see below) led us to focus on FAK1 as the potential target. Moreover, previous reports using competition binding approaches to assess inhibitor selectivity/potency have identified crizotinib as a potent inhibitor of FAK1 ([22] and HMS Library of Integrated Network-based Cellular Signatures PF02341066 KINOMEscan dataset 20033). Multiple lines of evidence from our work suggest that FAK1 is indeed the relevant target of crizotinib. First, our data confirm that crizotinib treatment inhibits the activation of FAK1, as determined by phosphorylation of tyrosine 397 which correlates with activation of this kinase [25, 26]. Second, treatment of *NF2*-null Schwann cells with small molecules inhibitors of FAK1 result in inhibition of cell proliferation in a dose-dependent manner, mimicking the outcomes of crizotinib treatment. Third, and most important, direct inhibition of FAK1 in two *NF2*-null Schwann cell lines, using siRNA or shRNA, recapitulates the phenotypes observed with crizotinib treatment and confirms that loss of FAK1 is incompatible with proliferation of these cells. Finally, the ability of crizotinib resistant FAK1 mutants to shift and increase the EC_50_ of crizotinib against SC4 cells suggests that the effects of crizotinib in these cells are mediated largely through FAK1 inhibition. While these studies do not exclude the possible contribution of other targets, the extent and concordance of the phenotypes observed suggest that inhibition of FAK1 is the major mechanism through which the effects of crizotinib are mediated.

FAK1 (coded by the *PTK2* gene) is a cytoplasmic tyrosine kinase shown to be upregulated and activated in a range of solid tumor types. FAK1 promotes cell proliferation, survival, motility and other functions, through kinase-dependent and independent mechanisms [27]. Recent studies in a panel of mesothelioma cells lines revealed that sensitivity to FAK1 inhibition was inversely-correlated to levels of Merlin expression. The proposed mechanism suggests Merlin loss leads to destabilization of cell:cell junctions, which causes the cells to become more reliant on cell-ECM interactions for survival and proliferation. As FAK1 is a critical effector of these proliferative/survival signals, the consequences of FAK1 inhibition in the context of NF2 loss are detrimental to the cells [18]. Other studies examining the relationship between Merlin and FAK1 in ovarian cancer cell identified a correlation between Merlin expression levels and sensitivity to FAK1 inhibitors, when cells were grown in an anchorage-independent manner. Intriguingly, stable knockdown of Merlin in resistant cells did not sensitize cells to FAK1 inhibition. This suggests that at least in ovarian cancer cell lines Merlin expression might predict responsiveness to FAK1 inhibitors, although a causal link between Merlin and FAK1 was not identified [17]. These recent findings, along with the data presented in our report, strongly implicate FAK1 as a critical effector and as a relevant target in the context of Merlin deficiency.

From a clinical standpoint, crizotinib represents an excellent candidate for evaluation in NF2. It is well tolerated and has already received FDA approval for the treatment of patients with metastatic non-small cell lung cancer that are positive for activating ALK fusions. In addition, crizotinib is being evaluated in phase I/II trials in children with relapsed/refractory solid tumors and primary CNS tumors such as neuroblastoma (NCT00939770). In addition to assessing the efficacy of crizotinib against NF2-deficient schwannomas, the activity of crizotinib against other *NF2*-null tumors, such as meningiomas and mesotheliomas, should be evaluated. While crizotinib was developed as an MET/ALK inhibitor, there are a number of inhibitors specifically developed against FAK1 that are currently in various stages of clinical trial. These include GSK2256098, PND-1186 (VS-4718), PF-271 (VS-6062) and defactinib (VS-6063) [27]. Once approved, these drugs could represent additional therapeutic options for *NF2*-null tumors and should thus be evaluated. Interestingly, our studies with PND-1186 and defactinib suggest a similar potency of these drugs compared to crizotinib. This suggests that the inhibitory capacity of crizotinib against FAK1 is sufficient to elicit a favorable effect. However, a true comparison of these drugs will ultimately require assessment in a clinical trial setting.

In summary, our studies identify an FDA approved drug, crizotinib, which shows *in vitro* and *in vivo* efficacy against *NF2*-null schwannomas. Furthermore, our studies identify the mechanism of action, through the inhibition of FAK1. Given the lack of curative options for NF2 and the favorable tolerability of this drug, crizotinib represents an excellent candidate for clinical testing in patients harboring NF2-deficient tumors.

## MATERIALS AND METHODS

### Cell culture conditions

The SC4 and HEI193 cell lines were obtained from The House Ear Institute and ATCC, respectively. The hSC2λ cells are a gift from Dr. Margaret Wallace (U. of Florida) [28]. All cell lines were authenticated by short tandem repeat (STR) DNA profiling (DDC Medical) (March 2015) and verified to be clean of mycoplasma contamination using a PCR based approach. Cells were grown in low-glucose DME, 10% fetal calf serum, 1× non-essential amino acids and 100 IU/ml penicillin-streptomycin. Transfections were performed with Lipofectamine 2000 (Invitrogen), following manufacturer's instructions.

### Western blot analysis

Protein extracts were prepared with RIPA lysis buffer (50 mM TRIS-HCl, pH 7.5, 1% Nonidet P-40, 0.25% sodium deoxycholate, 150 mM NaCl, 1 mM EGTA, 1 mM sodium orthovanadate, and 1 mM NaF). The following antibodies were used according to manufactures’ instructions: MET, FAK1 and FAK-pY397 (cat# 3127, 3285, 3283 - Cell Signaling Technology).

### RNAi-mediated knockdown of FAK

HEI193 and SC4 cells were transfected using Lipofectamine 2000 (Invitrogen, NY, USA), according to the manufacturer's instructions. Specifically HEI193 cells were transfected with siRNA duplexes targeting FAK1 [ID# SR303877A- GCAAUGGAGCCGAGUAUUA AAGGUCT and SR303877B- AGAAGAUACUUACA CCAUGCCCUCA] as well as a non-targeting siRNA control [ID # SR30004- CGUUAAUCGCGUAUAAUACG CGUAT] (Origene, Rockville, USA) at a concentration of 30 nM. SC4 cells were transfected with TRC mouse shRNA individual clones targeting FAK1 [ID # TRCN0000 023484 −5′-CGGTCCAATGACAAGGTATAT-3′ and TRCN0000023486–5′-CCAACCTTAATAGAGAAGAA A-3′] (GE Dharmacon, Lafayette, USA).

### CRISPR/Cas9 editing of MET

Guide sequences from exon 1 of the *MET* locus (g#1- GTTTACTGACATACGCGGCT and g#2- GTTCATCTCAGACTTCACTA) were cloned into the pX459 vector (Addgene). SC4 cells were transfected with 8 μg of plasmid into the SC4 cells using Lipofectamine 2000, according to manufacturer's instructions. Cells were selected for 48 hours in puromycin and individual clones were picked and expanded after 7 days. Clones were analyzed by western blotting to determine loss of MET expression.

### Cell proliferation assays

30,000 cells/well were plated in 12-well dishes in triplicate. At indicated time points, cells from individual wells were trypsinized and counted using a Coulter counter (Z1 series, Beckman Coulter). Cell growth media was replaced daily. For measurement of cell proliferation, cells were plated at the numbers indicated in the figures and BrdU Proliferation Assay (Millipore) was used according to the manufacturer's instructions. Statistical significance was determined by a two-tailed student's *t*-test. Each condition at each time point represents the mean of 3 experiments in triplicate for a total of 9 wells.

### Generation of FAK1 mutants and stable SC4 clones

The FAK1 mutant constructs were created by PCR amplification of FAK1 cDNA from the pLV-Neo-CD2-FAK1 (addgene #37013) and cloning into pcDNA3.1 using NheI and NotI sites (primer sequences Fak-Nhe1F 5′-aaaagctagcaaaATGGCAGCTGCTTACCTTGACC-3′, Fak-Not1R 5′-aaaagcggccgcaaaTCAGTGTGGTCTCG TCTGCC-3′). Site directed mutagenesis was performed using the Agilent Quick Change Site-Directed Mutagenesis Kit (cat# 200518-5). Primers were designed using the SDM design tool on the Agilent website. Primer sequences: FAKS509R: 5′-gcacacttggagagctgaggagatttttgcaagtaaggaaat-3′ and 5′-atttccttacttgcaaaaatctcctcagctctccaagtgtgc- 3′. FAKG563S: 5′ –ctggtgtcctcaaatgattgtgtaaaattaagcgactttggattatccc-3′ and 5′- gggataatccaaagtcgcttaattttacacaatcatttgaggacaccag-3′. Bacterial clones were isolated and sequence verified. SC4 Cells were transfected with 8 μg of plasmid DNA, using Lipofectamine 2000 (cat# 11668019), and were placed under G418 selection, 150 ug/ml until individual clones could be picked and expanded for experimentation.

### RNA sequencing data

RNA from SC4 cells was extracted using TRIZOL reagent. 5–10 μg of total RNA was used to isolate poly(A) RNA using the micropoly(A) purist kit (Ambion). The whole transcriptome library kit (Life Technologies) was used to prepare paired-end sequencing libraries. Briefly, 100 ng of poly(A)^+^ RNA was enzymatically fragmented to an average size of 150 nt, ligated to directional adapters and reverse-transcribed, and the cDNA was size-selected on denaturing 6% TBE-Urea polyacrylamide gels, amplified for 15 cycles with barcoded primers and purified using AmpPure XP beads. The resulting library was quantified using Bioanalyzer (Agilent) and Taqman (Life Technologies) assays. The barcoded library combined was used at a final concentration of 0.4 pM in emulsion PCR to link to the sequencing beads. For analysis, the sequencing reads in color-space were mapped to the mm9 genome using Tophat [29]. The number of reads falling into each gene defined in the RefSeq gene annotations was quantified using HTSeq-count [30]. Samples from three independent experiments were sequenced, combined, and analyzed to produce the final DESeq data. The RNA-Seq data is publicly available through the NCBI GEO database with accession number GSE61528.

### Determination of EC_50_s and dose response analysis

To determine the EC_50_ of crizotinib 2500 cells from each clone were plated, in quadruplicate, in a solid white cell culture 384-well plate. Crizotinib was added to the wells in a three-fold ten-point dilution series ranging from 12.5 μM to 0.0006 μM. Crizotinib activity was evaluated 24 hours after treatment using CellTiter-Glo (Promega, USA) as a read-out of viability. The EC_50_ values were calculated using Graphpad Prism.

### *In vivo* tumor models and imaging

All animal experiments were approved by the Scripps Institutional Animal Care and Use Committee and performed in accordance with relevant institutional and national guidelines. The use of the orthotopic tumor model was previously described [31]. Briefly, *Nf2*^−/−^ SC4 Schwann cells were transduced by lentiviruses carrying pLuc-mCherry and sorted by FACS. 5 × 10^4^ cells were transplanted into the sciatic nerve sheath of NOD/SCID mice (8 weeks of age) by intraneural injection. Tumor progression was monitored every 3 days by bioluminescence imaging (BLI) according to the manufacturer's instructions on an IVIS-200 system (Xenogen, San Francisco, CA).

### MIB chromatography

Cells were treated with 10 μM crizotinib for the indicated time points and tumors were harvested as previously described [16]. Protein lysates containing 3 mg of total protein each were brought to 1 M NaCl and passed through columns of inhibitor-conjugated beads (Bisindoylmaleimide-X, SB203580, Dasatinib, PP58, VI16832, and CTx-0294885) to isolate protein kinases. Kinase-bound inhibitor beads were washed with high- and low-salt buffers and 0.1% SDS before elution by boiling in 0.5% SDS with 1% β-mercaptoethanol in 0.1 M Tris-HCl (pH 6.8). Proteins were purified using chloroform/methanol extraction and resuspended in 50 mM HEPES (pH 8). Samples were digested overnight at 37°C using sequencing grade modified trypsin (Promega). Peptides were fractionated with Mini SCX spin columns and cleaned with PepClean C18 Spin Columns (Thermo Scientific) before MS analysis.

### MS analysis

MS and MS/MS data were acquired with a Q Exactive™ Hybrid Quadrupole-Orbitrap Mass SpectroMETer (Thermo) equipped with an EASY-nLC1000 nano UHPLC (Thermo). Each individual sample was resuspended in 20 μL of sample buffer (2% ACN, 0.1% TFA) and injected (5 μL) onto the Thermo Scientific EASY-Spray column (C18, 2 μM, 100 A, 75 μM X 25 cm). A linear gradient from 5% B (CH_3_CN + 0.1% formic acid) to 25% B in 150 minutes and then 25% B to 45% B in 15 minutes, followed by 45% B to 95% B in 5 minutes and pumping 95% of B for additional 10 minutes. MS and MS/MS data were analyzed by MaxQuant 1.5.0.0 with a UniProtKB database using label free quantifications.

### Statistical analysis

To assess the anti-tumor activity of crizotinib *in vivo*, the natural log-transformed flux readings were used to reflect tumor growth. The trends of tumor growth over time (followed up by days) were examined between control and treatment groups using a mixed-effect model with the random effect at the mouse level. A linear function, in days, was determined to provide the best fit without random slope on follow-up days. A likelihood ratio testing nested models (with versus without the interaction term of group and time) was used to examine if trends are significantly different overall between groups. The tumor weights were first examined by a Shapiro-Wilk test to assess data normality and a Variance ratio test was used to examine the equality of variances between two groups. The differences between the control and experimental cohorts were analyzed by a *t*-test with equal variances. Statistical analyses were performed using Stata 12.0 (StataCorp LP, Texas, USA).

## SUPPLEMENTARY MATERIALS






