# Supplementary material for: Crizotinib inhibits NF2-associated schwannoma through inhibition of focal adhesion kinase 1
Source: Oncotarget. 2016 Jun 23;7(34):54515–25. doi: 10.18632/oncotarget.10248 (PMC5342359; doi:10.18632/oncotarget.10248)
Supplement: Supplementary file 1 [file oncotarget-07-54515-s001.pdf]

Crizotinib inhibits NF2-associated schwannoma through inhibition of focal adhesion kinase 1

Supplementary Materials

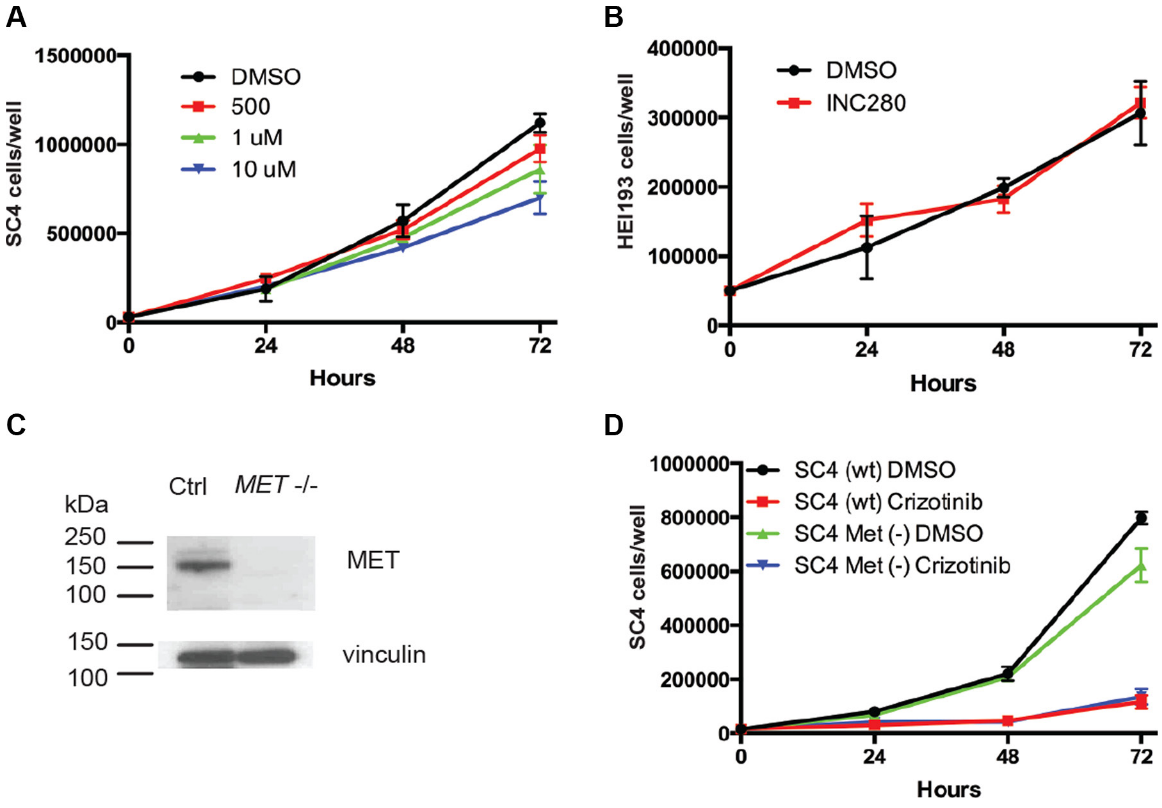

Supplementary Figure S1: Impact of MET inhibition on *NF2*-null Schwann cell proliferation. (**A**) SC4 cells treated with INC280 at the indicated concentrations or with 25% DMSO control, daily for 3 days. (**B**) HEI193 cells treated with INC280 (10 uM) or crizotinib (10 uM) or with 25% DMSO control, daily for 3 days. (**C**) Western blot analysis of MET expression in SC4 and $SC4^{MET(-)}$ (MET knockout) cells. Tubulin was used as a loading control. (**D**) SC4 or $SC4^{MET(-)}$ cells treated with crizotinib (10 uM) or with 25% DMSO control, daily for 3 days. In all counting experiments cell numbers were scored daily and each time point was done in triplicate. The data shown is the mean of 3 independent experiments. Error bars = SD.

Supplementary Table S1: Relative expression of kinases in SC4 cells. See Supplementary_Table_S1

Supplementary Table S2: Activity based protein profiling in SC4 cells treated with Crizotini. See Supplementary_Table_S2
